# Evaluation of the high-throughput RespiV *PlexPlus* multiplex assay compared to BioFire Respiratory Panel 2.1 *plus*

**DOI:** 10.1128/spectrum.00019-26

**Published:** 2026-05-11

**Authors:** Todd M. Pryce, Erin J. Haygarth, Angelica S. Lacsina, Riley D. Oorschot, Edward Raby

**Affiliations:** 1 PathWest Laboratory Medicine WA, Department of Clinical Microbiology, Fiona Stanley Hospital, Murdoch, Western Australia, Australia; MultiCare Health System 14458, Tacoma, Washington, USA

**Keywords:** BioFire, Respiratory Panel 2.1 *plus*, *PlexPlus*, RespiV, respiratory pathogens

## Abstract

During the 2023 Australian winter respiratory virus season, 2,000 clinical samples were prospectively tested over 47 days (July–August 2023) using the BioFire Respiratory Panel 2.1 *plus* (RP2.1+) assay, which detects 19 viral and 4 bacterial targets. These samples were retrospectively tested with the RespiV *PlexPlus* (RespiV) assay, which detects 14 viral targets. RespiV demonstrated high overall percentage agreement with RP2.1+ (99.2%), with a negative percent agreement exceeding 99% for the majority of shared targets. Positive percent agreement was more variable across targets, with more positive discordant results for high-prevalence pathogens. Positive discordant results had higher quantification cycle values (median 37.2) than concordant positives (median 27.8), consistent with near-limit-of-detection inter-assay discordance. Discordant results for influenza A virus, influenza B virus, respiratory syncytial virus, and severe acute respiratory syndrome coronavirus 2 (SARS-CoV-2) were retested with Xpert Xpress Flu/RSV and Xpress SARS-CoV-2 to generate a consensus result. RP2.1+ and RespiV showed 99.6% overall agreement with the consensus result for these targets. RespiV detected 14 additional influenza A-positive samples, all confirmed by amplicon sequencing. RespiV also identified 19 enterovirus-positive samples, 17 of which were RP2.1+ rhinovirus/enterovirus positive. Conversely, RP2.1+ detected pathogens not included in the RespiV panel, such as 79 non-SARS-CoV-2 coronaviruses and 1 *Chlamydophila pneumoniae*, with 62 samples negative across all RespiV targets. In summary, both assays show a high degree of concordance for all shared targets. RP2.1+ offers rapid test turnaround and broader pathogen coverage, making it valuable for urgent diagnostics, while RespiV provides higher throughput and is well-suited for centralized, high-volume testing environments.

**IMPORTANCE:**

Rapid detection and differentiation of severe acute respiratory syndrome coronavirus 2 (SARS-CoV-2) and other treatable respiratory pathogens are critical for guiding therapy and infection control. Sample-to-answer multiplex panels, such as the BioFire Respiratory Panel 2.1 *plus*, allow simultaneous detection of multiple pathogens. These assays are fast, user-friendly, and support random-access testing for small batches, but they are costly and limited in throughput by instrument availability. Conversely, conventional multiplex assays like RespiV *PlexPlus* are slower and more labor-intensive for small batches but optimized for large batch processing on standard molecular diagnostic platforms. They offer higher throughput with lower consumable costs, making them suitable for centralized laboratories. Recent innovations in both sample-to-answer and conventional multiplexing technologies have expanded detectable targets, with comparable technical performance between formats. Ultimately, the optimal choice depends on healthcare system resources and testing demands.

## INTRODUCTION

Respiratory infections significantly impact global health and are a major cause of hospital admission (1, 2). In 2019, the global burden of upper respiratory infections was estimated at 17.2 billion cases (95% uncertainty interval: 15.4–19.3 billion) (3). The World Health Organization (WHO) reported 14.9 million deaths from severe acute respiratory syndrome coronavirus 2 (SARS-CoV-2) between 2020 and 2021 (4). Consequently, diagnostic testing for SARS-CoV-2 and other respiratory pathogens has surged. Timely diagnosis of respiratory infections is crucial for patient management, optimizing treatment, allocating bed spaces appropriately, and preventing transmission. The initial diagnosis is based on clinical suspicion of acute respiratory illness (ARI), but a definitive diagnosis requires laboratory testing. Commercial multiplex PCR panel testing systems, or syndromic panels, have been deployed to improve test turnaround time compared to centralized laboratory-based PCR testing, with increasing numbers of pathogen targets (5, 6).

**Fig 1:**
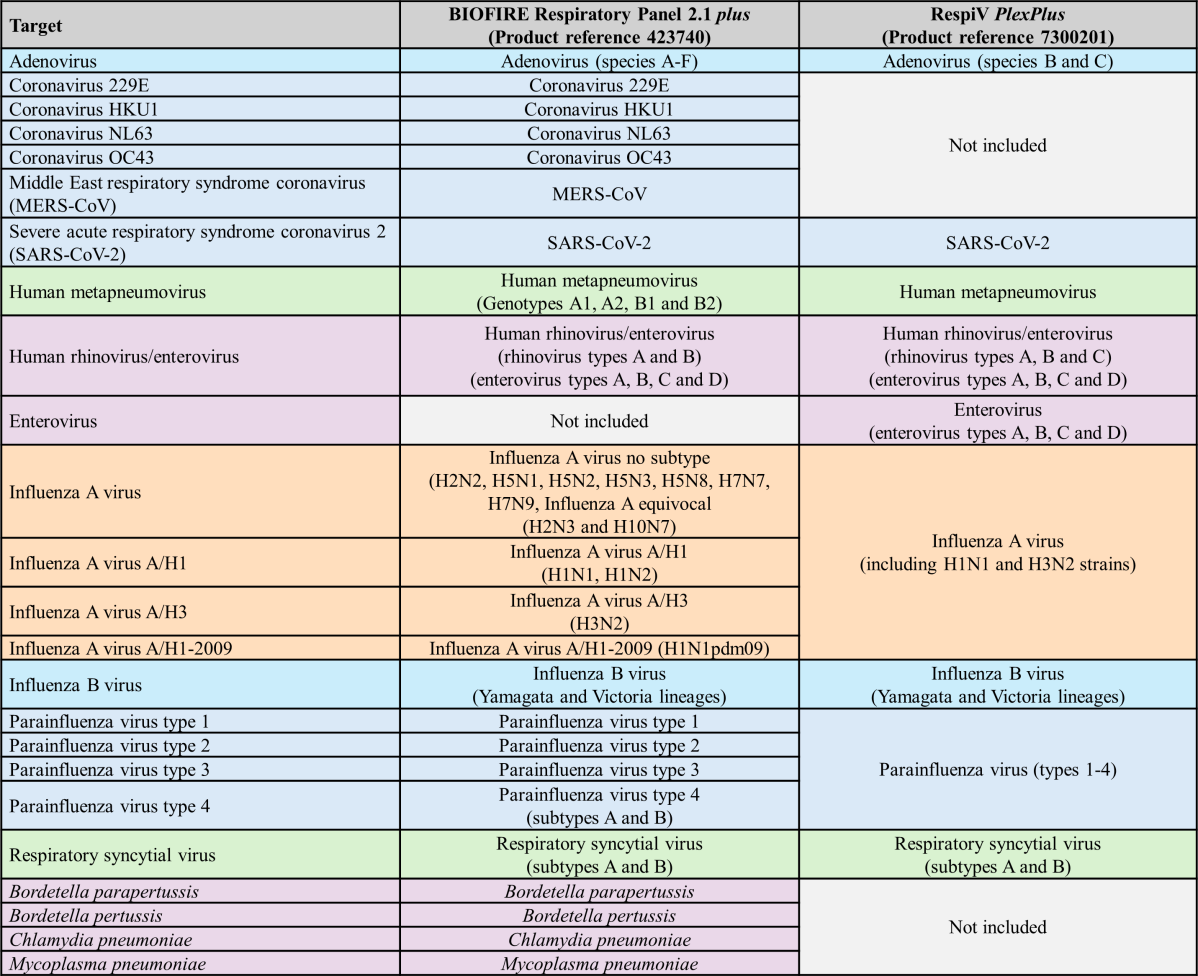
Summary of the targets and specificity claims for BioFire Respiratory Panel 2.1 *plus* and RespiV *PlexPlus.*

One example of a rapid sample-to-answer test is the BioFire Respiratory Panel 2.1 *plus* (RP2.1+; bioMérieux, Marcy l'Etoile, France), which can detect multiple respiratory pathogens in approximately 45 min. As summarized in Fig. 1, RP2.1+ provides broad respiratory coverage (including several bacterial targets) but does not differentiate human rhinovirus (HRV) from enterovirus (EV). Due to the lack of a dedicated onsite molecular diagnostic testing laboratory, this assay is performed at our near-point-of-care satellite laboratory based at Royal Perth Hospital, Perth, Western Australia, to primarily service its emergency department (ED). Rapid respiratory PCR results (<3 h from collection) are reported to determine ARI status before medical ward admission or discharge from ED. While RP2.1+ offers rapid and broad pathogen detection, its limited throughput, high consumable cost, and the repetitive nature of sustained testing present ongoing operational challenges.

**Fig 2:**
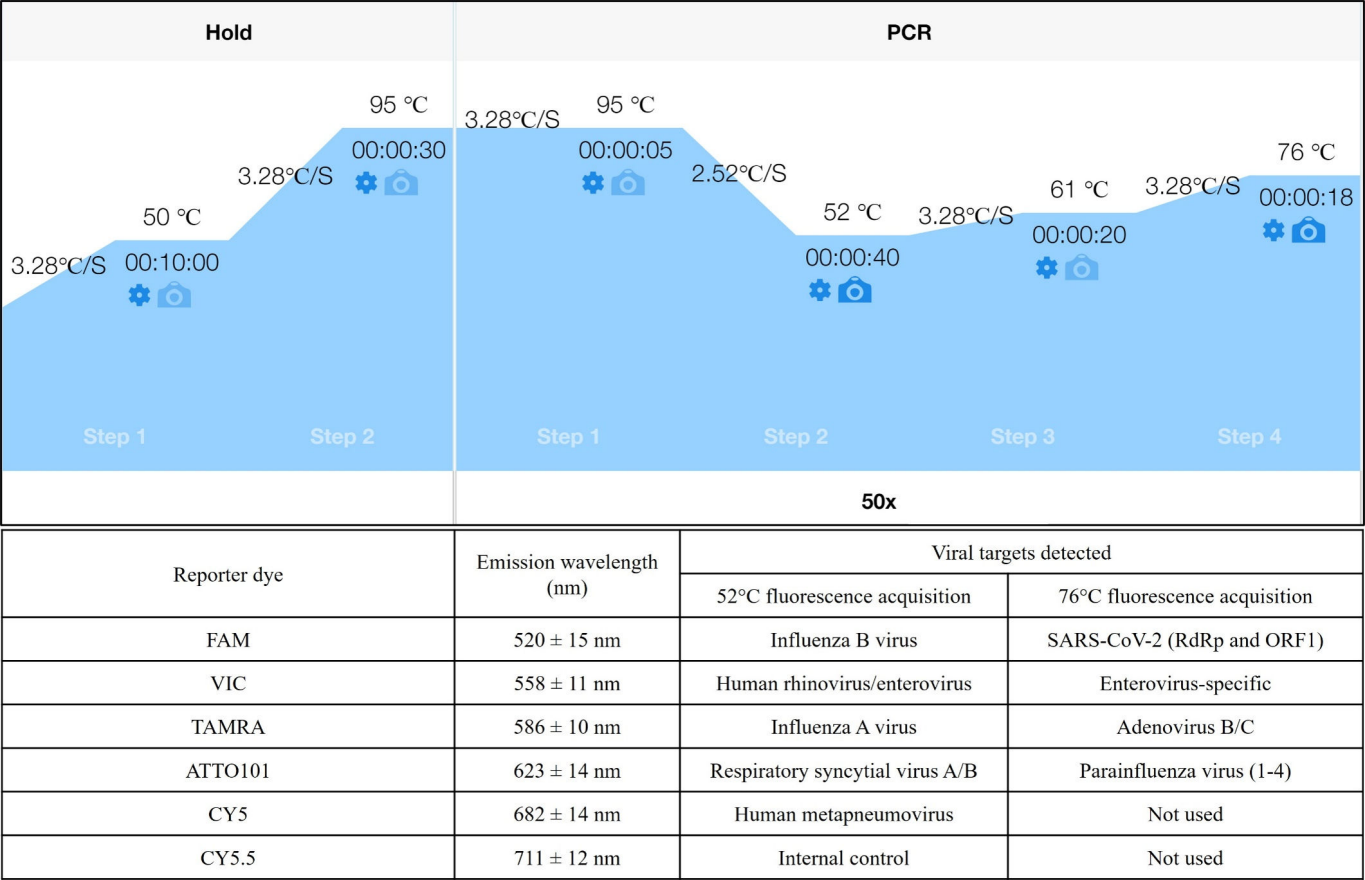
Thermal cycling conditions and targets detected using RespiV *PlexPlus*. Thermal cycling conditions for the QuantStudio 5 real-time PCR instrument, showing thermal cycling steps, hold temperatures (°C), hold times (hh:mm:ss), fluorescence acquisition (bold camera icon), and the number of PCR cycles. The reporter dye, emission wavelength (nm), and the viral targets detected at each temperature are shown.

Conventional multiplex assays offer an alternative approach, enabling batched, high-throughput testing on standardized molecular platforms. At our centralized laboratory, we have used a batched PlexPCR RespiVirus (SpeeDx, Eveleigh, NSW, Australia) and PlexPCR SARS-CoV-2 (SpeeDx) workflow (7), which requires three amplification wells per sample to provide broad viral coverage within a routine shift. Recently, multiplexing capability has been improved by SpeeDx with the RespiV *PlexPlus* assay (RespiV) (8). This research-use-only assay detects the same viral targets as PlexPCR in a single well and adds EV with HRV/EV differentiation (Fig. 1 and 2), which is a key point of difference compared to RP2.1+. RespiV uses next-generation qPCR technology (*PlexPlus*), which detects multiple targets within each optical channel using universal, low- and high-temperature probes, allowing more targets per well in standard 96- or 384-well thermal cyclers while maintaining throughput.

The goal of this evaluation was to assess RespiV as a replacement for the 3-well PlexPCR workflow, aiming to enhance laboratory efficiency and clinical utility. Here, we retrospectively tested 2,000 samples with RespiV, which had previously been tested with RP2.1+. The concealed proprietary algorithm behind RP2.1+ limits identification of potentially false-positive results, or those around the lower limit of detection, which complicates interpretation of discordant results. Therefore, samples with discordant results for the most clinically important shared targets, which include influenza A virus (FluA), influenza B virus (FluB), respiratory syncytial virus (RSV), and SARS-CoV-2, were further tested with Xpert Xpress Flu/RSV or Xpress SARS-CoV-2 (Xpert; Cepheid, Sunnyvale, CA, USA) to define a consensus comparator result—the Xpert tests have consistently shown high accuracy and a low limit of detection for these targets (9, 10). In addition, FluA discordant samples were investigated with RespiV amplicon sequencing. We also tested RP2.1+, Xpert, and RespiV using the 1^st^ WHO International Standard (IS) for SARS-CoV-2 RNA to compare the analytical sensitivity of SARS-CoV-2 detection and to standardize the quantification cycle (*C_q_*) analysis to assist with discordant result interpretation. In summary, we present the first laboratory evaluation of RespiV and assess its performance compared to RP2.1+, highlighting the high-throughput capability of this novel single-well multiplex assay.

## MATERIALS AND METHODS

### Clinical specimens and RP2.1+ testing

Two thousand naso-oropharyngeal swabs were collected from the ED and hospitalized patients within our hospital network. The majority of swabs (*n* = 1,992) were received as dry flocked swabs (Copan P/N 520CS01) via pneumatic chute from the ED and inoculated into 3 mL of virus transport medium (VTM) without delay, as described previously (7). The remaining samples (*n* = 8) were collected in 3 mL of universal transport medium (Copan UTM-RT media, Brescia, Italy) at the point of care. Samples were processed in a biosafety cabinet with operators wearing appropriate personal protective equipment. Each sample was prepared individually, and the workspace was cleaned between specimens, following the manufacturer’s instructions. RP2.1+ testing was conducted according to the manufacturer’s guidelines. RP2.1+ sample processing and testing occurred 24 h per day. Over a 47-day period from July to August 2023, 2,000 consecutively collected samples were prospectively tested with RP2.1+ as part of routine diagnostic testing during the Australian winter respiratory virus season (June–September 2023), when there was co-circulation of influenza and SARS-CoV-2. After testing, residual VTM samples were initially stored at −20℃ for 1 week and then transferred to −80°C in an accessioned storage system. Personal and identifying sample metadata were removed and replaced with a laboratory-generated identifier to allow ascertainment of repeat testing.

### RespiV testing

All samples were retrospectively tested with RespiV over a 12-day period in May 2024. For operational efficiency, run sizes of 90 were tested, which included 87 samples retrieved from −80°C storage, 1 uninoculated VTM, and 2 positive RespiV controls (controlling all targets). A total of 23 batched runs were performed. RespiV testing was performed following the recommendations by the manufacturer. Briefly, samples were extracted using the MagNA Pure 96 instrument (MP96; Roche) with Pathogen Universal 200 protocol (version 4.0). A 200 μL aliquot of the sample was used and extracted with the MP96 DNA and Viral NA Small Volume Kit (Roche) and eluted in 50 μL. A 20 μL aliquot of the RespiV internal control was added to each sample using the MP96 internal control addition protocol. The internal control consisted of 36 µL of RespiV IC RNA in 3,564 µL of phosphate-buffered saline. All samples were immediately returned to −80°C storage for further discordant testing if required. RespiV was performed immediately following nucleic acid extraction using 10 μL of RespiV master mix dispensed into MicroAmp EnduraPlate 96-well PCR plates (Applied Biosystems, ThermoFisher, Waltham, MA, USA) in the pre-PCR area using the QIAgility liquid handler (QIAGEN, Hilden, Germany). A 10 μL volume of nucleic acid template was added to each well using the MP96 volume transfer protocol. Amplification, detection, and analysis were performed using the QuantStudio 5 instrument (Applied Biosystems, ThermoFisher). The thermal cycling conditions and temperature-dependent fluorescence acquisitions for each target are shown in Fig. 2. Result analysis was conducted using a combination of relative and baseline threshold analysis. It should be noted that RespiV HRV/EV-positive samples that are EV negative are recorded as HRV positive. However, the presence of HRV cannot be excluded for those samples testing positive for EV. All qualitative results, including the *C_q_* values for all positive targets and the internal control, were recorded, and these are shown in the supplemental material. All PCR plates were stored at −20°C for amplicon sequencing testing if required.

### Comparator testing and discrepant analysis

RP2.1+ results were retrieved from the laboratory information system and used as the comparator method for the evaluation of the RespiV assay, designated as the candidate method. Agreement between methods was assessed for each shared pathogen target. Result pairs were classified as concordant positive (candidate positive/comparator positive), concordant negative (candidate negative/comparator negative), positive discordant (candidate positive/comparator negative), or negative discordant (candidate negative/comparator positive), without inferring clinical truth in the absence of an established reference standard.

To further characterize discrepancies for FluA, FluB, RSV, and SARS-CoV-2, specimens exhibiting discordant results between methods were retrieved from −80°C storage and retested with Xpert in June 2024, following the manufacturer’s instructions. A consensus reference result (consensus result) was assigned for each target as follows: the target was classified as positive if two of the three methods reported detection, and negative if two of the three methods reported no detection. This consensus result was subsequently used as the comparator for separate evaluations of the RespiV and RP2.1+ assays, each treated as a candidate method.

### RespiV influenza A sequencing

All samples that were RespiV FluA-positive, which were defined as FluA-negative (according to the consensus result), were identified. These sample amplicons were retrieved from the PCR plates stored at −20°C. The amplicons were diluted 1:100 with water and re-amplified using a nested forward primer and the RespiV reverse primer. Non-template controls were also included to exclude contamination of our amplicon sequencing approach. Amplification conditions used were 95°C for 2 min, followed by 50 cycles of 95°C for 15 s and 60°C for 60 s. Amplicons were sequenced with the nested forward primer and the RespiV reverse primer using Sanger sequencing (Australian Genome Research Facility, Westmead, Sydney, NSW, Australia). Sequences were searched against the GenBank core nucleotide database using BLAST and aligned to influenza A virus GenBank OQ203985 (A/Wisconsin/67/2022/H1N1).

### Quantitative SARS-CoV-2 standard preparation and testing

To compare the analytical sensitivity of RP2.1+, RespiV, and Xpert for SARS-CoV-2 detection and to standardize *C_q_* analysis for RespiV and Xpert, quantitative standards were prepared from the 1^st^ WHO International Standard for SARS-CoV-2 RNA (SARS-CoV-2 IS, NISBSC code 20/146), supplied as 7.70 log_10_ IU/mL (National Institute for Biological Standards and Control, Hertfordshire, UK). The standard was reconstituted with 0.5 mL of phosphate-buffered saline following the manufacturer’s instructions. Once reconstituted, the standard was 10-fold serially diluted in a naso-oropharyngeal matrix. This matrix consisted of pooled naso-oropharyngeal samples from samples previously tested as negative for SARS-CoV-2 using Xpert. Seven standards were prepared over the range of 0.70–6.70 log_10_ IU/mL. Each standard was tested with RP2.1+ and Xpert and in duplicate with RespiV using the same amplification and detection lot numbers used for patient samples. The mean *C_q_* value was used to calculate the standard curve and regression for each assay.

### Data and statistical analysis

Agreement between each candidate assay and its comparator was evaluated per shared pathogen target in accordance with Clinical and Laboratory Standards Institute protocol EP12-A2 (11). Asymmetric clinical agreement metrics—positive percent agreement (PPA), negative percent agreement (NPA), and overall percent agreement (OPA)—were calculated and are presented with 95% Wilson score confidence intervals. Because neither assay can be assumed to represent a definitive reference method, the symmetric chance-corrected agreement statistic Cohen’s kappa (*κ*) was calculated to provide an additional measure of inter-method agreement. Cohen’s kappa is presented with 95% bias-corrected and accelerated bootstrap confidence intervals based on 10,000 resamples. To aid interpretation of *κ* in settings with potential imbalance or systematic bias, the prevalence index (PI), bias index (BI), and prevalence-and bias-adjusted kappa (PABAK) were calculated following Byrt et al. (12). PABAK is presented as a point estimate only; its uncertainty is fully represented by the OPA confidence interval through the identity PABAK = 2 × OPA − 1. All analyses were conducted using R version 4.5.2 (13), employing the *boot* package for bootstrap resampling (14).

## RESULTS

### Agreement between RespiV and RP2.1+

A summary of agreement metrics for each paired pathogen target is shown in Table 1. Overall, RespiV showed 99.2% OPA with RP2.1+. NPA exceeded 99% for all individual targets with the exceptions of HRV/EV (98.3%) and SARS-CoV-2 (98.6%). PPA was more variable, ranging from 88.3% (SARS-CoV-2) to 96.5% (FluB) across the majority of targets. Human adenovirus was an outlier, showing low PPA (50.0%, 95% CI: 23.7%–76.3%) with low certainty of the estimate in the context of a low number of positive (*n* = 12) calls. Human metapneumovirus demonstrated perfect agreement (PPA 100%) across all nine positive samples.

**TABLE 1:** Agreement between RespiV as candidate and RP2.1+ as comparator for paired pathogen targets^*j*^

Pathogen	CP	PD	ND	CN	PPA (%) (95% CI^*h*^)	NPA (%) (95% CI^*h*^)	OPA (%) (95% CI^*h*^)	Cohen's Κ (95% CI^*i*^)	PABAK	PI	BI
Influenza A virus	69^*a*^	14	6^*b*^	1,911	92.0 (83.6, 96.3)	99.3 (98.8, 99.6)	99.0 (98.5, 99.4)	0.87 (0.80, 0.92)	0.98	−0.92	0
Influenza B virus	55	2	2	1,941	96.5 (88.1, 99.0)	99.9 (99.6, 100)	99.8 (99.5, 99.9)	0.96 (0.91, 0.99)	>0.99	−0.94	0
Respiratory syncytial virus	81	5	6	1,908	93.1 (85.8, 96.8)	99.7 (99.4, 99.9)	99.5 (99.0, 99.7)	0.93 (0.88, 0.96)	0.99	−0.91	0
SARS-CoV-2	83	18	11	1,888	88.3 (80.2, 93.3)	99.1 (98.5, 99.4)	98.6 (97.9, 99.0)	0.84 (0.78, 0.89)	0.97	−0.9	0
Human adenovirus	5	2	5	1,988	50.0 (23.7, 76.3)	99.9 (99.6, 100)	99.7 (99.3, 99.8)	0.59 (0.25, 0.84)	0.99	−0.99	0
Human metapneumovirus	9	0	0	1,991	100 (70.1, 100)	100 (99.8, 100)	100 (99.8, 100)	1.00^*g*^	1	−0.99	0
Human parainfluenza virus (1–4)	47^*c*^	2	5^*d*^	1,946	90.4 (79.4, 95.8)	99.9 (99.6, 100)	99.7 (99.3, 99.8)	0.93 (0.85, 0.97)	0.99	−0.95	0
Human rhinovirus/enterovirus	173^*e*^	31^*f*^	11	1,785	94.0 (89.6, 96.6)	98.3 (97.6, 98.8)	97.9 (97.2, 98.4)	0.88 (0.84, 0.91)	0.96	−0.81	0.01
Overall	522	74	46	15,358	91.9 (89.4, 93.9)	99.5 (99.4, 99.6)	99.2 (99.1, 99.4)	0.89 (0.87, 0.91)	0.99	−0.93	0

^*a*^ FluA H3 (*n* = 17), FluA H1-2009 (*n* = 50), and FluA no subtype (*n* = 2).

^*b*^ FluA H3 (*n* = 1), FluA H1-2009 (*n* = 3), FluA no subtype (*n* = 1), and FluA equivocal (*n* = 1).

^*c*^ HPIV1 (*n* = 3), HPIV2 (*n* = 1), HPIV3 (*n* = 39), and HPIV4 (*n* = 4).

^*d*^ HPIV3 (*n* = 3) and HPIV4 (*n* = 2).

^*e*^ Seventeen samples were RespiV EV-positive that were RP2.1+ HRV/EV-positive.

^*f*^ Two samples were RespiV EV-positive that were RP2.1+ HRV/EV-negative.

^*g*^ Perfect agreement (no discordant results); confidence intervals are not applicable.

^*h*^ Wilson score confidence interval.

^*i*^ Bias-corrected and accelerated bootstrap confidence intervals.

^*j*^ CP, concordant positive; PD, positive discordant (RespiV positive/RP2.1+ negative); ND, negative discordant (RespiV negative/RP2.1+ positive); CN, concordant negative; PPA, positive percent agreement; NPA, negative percent agreement; OPA, overall percent agreement; PABAK, prevalence‑adjusted bias‑adjusted kappa; PI, prevalence index; BI, bias index; and CI, confidence interval.

Overall, there were more positive discordant (*n* = 74; RespiV positive, RP2.1+ negative) than negative discordant results (*n* = 46; RespiV negative, RP2.1+ positive). Discordant results were concentrated among HRV/EV (*n* = 42), SARS-CoV-2 (*n* = 29), and FluA (*n* = 20).

From the supplemental material, positive discordant results were associated with substantially higher *C_q_* values (median 37.2, interquartile range 36.45–38.55) compared to concordant positive results (median 27.8, interquartile range 23.4–32.8).

As shown in Table 1, a negative correlation between PPA and the PI was observed across all targets (except human adenovirus), i.e., PPA reduced with increasing pathogen prevalence. The BI was zero for all targets except HRV/EV (BI 0.01), reflecting a modest increase in HRV/EV detections by RespiV. Of 19 EV-specific results detected by RespiV, 17 were also HRV/EV positive by RP2.1+. From 75 RP2.1+ FluA-positive results, 18 were FluA H3 (24%), 53 were FluA H1-2009 (71%), 4 were FluA no subtype (5%), and 1 was FluA equivocal. The majority of parainfluenza detections were HPIV3 (42/52, 81%).

### RP2.1+ and RespiV agreement to the consensus result

Sixty-nine discordant results from the primary analysis (20 FluA, 4 FluB, 11 RSV, and 29 SARS-CoV-2) were retested by Xpert to assign a consensus result. A summary of the RP2.1+ and RespiV results compared to the consensus result is shown in Table 2. Both assays achieved 99.6% overall agreement with the consensus result, with marginally higher concordant positive results (candidate positive, consensus positive) observed for RespiV (*n* = 300) than RP2.1+ (*n* = 295), primarily due to increased SARS-CoV-2 concordant positive results (94 and 89, respectively). Higher numbers of positive discordant (candidate positive, consensus negative) results were observed for RespiV (*n* = 27) than RP2.1+ positive discordant results (*n* = 18), primarily due to increased numbers of Flu A RespiV positive discordant results. Negative discordant results for SARS-CoV-2 (candidate negative, consensus positive) were marginally higher for RP2.1+ (*n* = 11) than RespiV (*n* = 6). There were no negative discordant results for RSV.

**TABLE 2:** Agreement of positive and negative RP2.1+ and RespiV results for influenza A virus, influenza B virus, respiratory syncytial virus, and SARS-CoV-2, compared to the consensus result^*b*^

Pathogen consensus result^*a*^	Assay	CP	PD	ND	CN	PPA (95% CI)	NPA (95% CI)	OPA (95% CI)
Influenza A virus	RP2.1+	70	5	0	1,925	100.0 (94.8–100.0)	99.7 (99.4–99.9)	99.8 (99.4–99.9)
	RespiV	69	14	1	1,916	98.6 (92.3–99.7)	99.3 (98.8–99.6)	99.3 (98.8–99.5)
Influenza B virus	RP2.1+	55	2	1	1,942	98.2 (90.6–99.7)	99.9 (99.6–100.0)	99.9 (99.6–99.9)
	RespiV	56	1	0	1,943	100.0 (93.6–100.0)	99.9 (99.7–100.0)	100.0 (99.7–100.0)
Respiratory syncytial virus	RP2.1+	81	6	0	1,913	100.0 (95.5–100.0)	99.7 (99.3–99.9)	99.7 (99.3–99.9)
	RespiV	81	5	0	1,914	100.0 (95.5–100.0)	99.7 (99.4–99.9)	99.8 (99.4–99.9)
SARS-CoV-2	RP2.1+	89	5	11	1,895	89.0 (81.4–93.7)	99.7 (99.4–99.9)	99.2 (98.7–99.5)
	RespiV	94	7	6	1,893	94.0 (87.5–97.2)	99.6 (99.2–99.8)	99.4 (98.9–99.6)
Overall	RP2.1+	295	18	12	7,675	96.1 (93.3–97.8)	99.8 (99.6–99.9)	99.6 (99.5–99.7)
	RespiV	300	27	7	7,666	97.7 (95.4–98.9)	99.6 (99.5–99.8)	99.6 (99.4–99.7)

^*a*^ All RP2.1+ and RespiV discordant results were tested with Xpert to define a consensus result (as described in Materials and Methods).

^*b*^ CI, confidence interval; CP, concordant positive; PD, positive discordant (candidate positive/consensus negative); ND, negative discordant (candidate negative/consensus positive); CN, concordant negative; PPA, positive percent agreement; NPA, negative percent agreement; and OPA, overall percent agreement.

For SARS-CoV-2 (from the supplemental material), 17 discordant samples from the primary analysis were Xpert-positive (N2 target, *C_q_* 36.0–44.3), while 12 were negative; 7 RespiV-positive/Xpert-negative samples had late *C_q_* values (36.7–39.1). For FluA, 1 sample was Xpert-positive (*C_q_* 39.2), 19 were negative; 14 RespiV-positive/Xpert-negative samples had late *C_q_* values (37.0–41.6). RSV and FluB showed mostly negative Xpert results, with a few RespiV positives (*C_q_* 34.1–36.8). Overall, Xpert supported RP2.1+ in 53% and RespiV in 47% of cases.

### RespiV discordant FluA testing and sequencing

Fourteen RespiV FluA-positive samples (consensus negative) were sequenced. Table S1 shows the original and repeat RespiV FluA *C_q_* values, target sequence length, and percent identity compared to influenza A (GenBank OQ203985). All showed FluA-specific amplicons (95.4%–97.2% identity). Also shown is a matrix table comparing these sequences to influenza A (GenBank OQ203985) and to each other (Fig. S1). The percentage match (bottom diagonal) and the number of mismatches (upper diagonal) are shown. Five different sequence contigs were observed (A–E). All results were considered detection at the lower limit of detection based on the late *C_q_* values (37.0–41.6). Samples with concomitant pathogens detected from original RP2.1+ and RespiV testing, including the results of repeat RespiV testing, are described in the footnote of Table S1. One sample remained FluA-positive on repeat RespiV testing; all others were negative, with one insufficient for retesting. Eight samples were originally positive for additional targets on both RP2.1+ and RespiV. On repeat RespiV testing, these co-detected targets were consistently positive with similar *C_q_* values. All other samples retested negative for all targets.

### Additional positive RP2.1+ targets not detected by RespiV

Sixty-two samples tested positive for a respiratory pathogen with RP2.1+ where RespiV reported a negative result (none detected). Overall, RP2.1+ detected 79 non-SARS-CoV-2 coronavirus occurrences and 1 detection of *Chlamydophila pneumoniae* from 76 samples. The summary table showing these results compared to RespiV results is shown in Table 3.

**TABLE 3:** Additional positive RP2.1+ targets not represented in the RespiV panel, showing all results for both assays according to the number of samples tested

Number of samples detected (*n* = 76)	RP2.1+ results		RespiV results
	Targets detected not represented in RespiV	Other targets co-detected represented in RespiV	
33	Coronavirus HKU1	No additional targets detected	No pathogens detected
13	Coronavirus NL63	No additional targets detected	No pathogens detected
10	Coronavirus OC43	No additional targets detected	No pathogens detected
3	Coronavirus HKU1 and Coronavirus OC43	No additional targets detected	No pathogens detected
3	Coronavirus HKU1	Rhinovirus/enterovirus	Rhinovirus
3	Coronavirus OC43	Rhinovirus/enterovirus	Rhinovirus
2	Coronavirus HKU1	Influenza B	Influenza B
1	*Chlamydophila pneumoniae*	Rhinovirus/enterovirus	Rhinovirus
1	Coronavirus 229E	Influenza A	Influenza A
1	Coronavirus NL63	Influenza A	Influenza A
1	Coronavirus OC43	Parainfluenza 4	Parainfluenza virus (1–4)
1	Coronavirus OC43	Respiratory syncytial virus	Respiratory syncytial virus
1	Coronavirus HKU1	SARS-CoV-2	SARS-CoV-2
1	Coronavirus OC43	Respiratory syncytial virus	No pathogens detected
1	Coronavirus OC43	Influenza A	No pathogens detected
1	Coronavirus HKU1 and Coronavirus NL63	No additional targets detected	No pathogens detected

### Multiple target detection

Limiting analysis to targets common to both assays, both RP2.1+ and RespiV detected three targets in one sample each and two targets in 26 and 31 samples, respectively, with RespiV more often detecting dual infection with HRV/EV and a second pathogen (detailed in supplemental material). Across all targets, RP2.1+ detected three targets in a single sample and two targets in 42 samples, with the increase primarily due to dual infection with seasonal coronaviruses and a second pathogen.

### Quantitative SARS-CoV-2 standard preparation and testing

The results for the SARS-CoV-2 quantitation standards for RP2.1+, RespiV, and Xpert are shown in Tables S2.1, S2.2, and S2.3. RespiV and RP2.1+ detected all standards to 2.70 log_10_ IU/mL; RespiV and Xpert detected one replicate at 1.70 log_10_ IU/mL. RespiV showed *R*² = 0.9968; Xpert *R*² = 0.9976–0.9986.

## DISCUSSION

This study evaluated the performance of the RespiV assay by retrospectively testing 2,000 clinical samples previously tested with RP2.1+, aiming to transition from the existing PlexPCR workflow. The strength of this evaluation lies in the large sample size, blinded retesting, and use of a third comparator assay (Xpert) to establish consensus results for key targets. In contrast, other investigations comparing BioFire to conventional respiratory multiplex PCR panels—capable of multiple target detection in a single fluorescence channel—tested only a limited number of samples and did not employ a third method to establish a consensus result (15). Consequently, those investigations were unable to accurately assess true positives and true negatives when comparing the Seegene Allplex Respiratory Panel 1, 2, 3 (Seegene Inc., Songpa-gu, Seoul, Korea) to BioFire. Although the Seegene assay includes coverage of the common coronaviruses in panel 3, it remains a multi-well format assay with lower throughput compared to RespiV.

Both RP2.1+ and RespiV demonstrated high agreement for all targets common to both assays. Except for human metapneumovirus, discordant results were observed for all other pathogens. These discordant results may be due to differences in species or virus type coverage or low levels of virus. For example, RP2.1+ reports coverage of human adenovirus species A–G, whereas RespiV targets only B and C. In addition, the genetic diversity within the Picornaviridae family (HRV and EV) makes detection of specific HRV and EV types a persistent challenge in clinical virology and public health surveillance (16). We were not able to resolve apparent systematic bias in HRV/EV metrics, which could indicate either increased sensitivity or decreased specificity of the RespiV assay for this target.

Discordance occurred most frequently with the highest prevalence pathogens. When prevalent, it is likely that a broader range of viral loads will be present across the sampled population, increasing the number approaching the limit of detection and inter-assay discordance. Consistent with this, discordant samples were observed to have higher *C_q_* values than concordant positives. These factors combined may account for many of these discordant results.

The highest proportion of discordant results when compared to the consensus result was observed for FluA, with 14 additional RespiV FluA-positive samples identified. Due to cost implications, we did not repeat FluA discordant samples with RP2.1+. Notably, eight of these samples were positive for one or more other targets. Initially, cross-talk between target acquisition temperatures and cross-talk between fluorophores was speculated, but assay controls and single detections of the same targets in other samples did not support this. To investigate further, all discordant FluA samples with sufficient residual volume were retested with RespiV from the original stored sample. Only one sample tested positive for FluA upon retesting with RespiV. However, for those samples where additional targets were co-detected, nearly all tested positive for the same targets upon repeat testing, thereby ruling out sample loading errors. Well-to-well contamination was also considered; however, analysis of the runs, including the run number and sample location compared to other influenza-A positive samples on the run (data not shown), did not support this. Additionally, we used automation for PCR plate set-up, extraction, and template transfer, essentially reducing the likelihood of laboratory contamination. Intrigued, we retrospectively investigated if these patients had follow-up repeat testing with RP2.1+, Xpert, or PlexPCR. One sample (Table S1: sample 1,835) was positive for FluA on our routine PlexPCR assay from a follow-up sample collected 7 days later (data not shown). No other patients had repeat follow-up testing. To exclude assay non-specificity, all the RespiV FluA-positive amplicons were sequenced and found to be influenza A-specific. Five distinct amplicon sequences were identified—while amplicon contamination cannot be entirely excluded, this heterogeneity is most consistent with low-level target originating from patient sample. We conclude that failure to re-detect FluA with RespiV or Xpert upon repeat testing is most likely attributable to a low viral load copy number, as indicated by the *C_q_* values, in combination with RNA degradation caused by long-term storage, repeat freeze-thaw cycles, and retesting up to 12 months later. As demonstrated in the analytical sensitivity assessment for SARS-CoV-2, RespiV may also exhibit greater sensitivity than RP21+ for FluA detection. Ongoing investigations using several FluA H3 and H1-2009 standards aim to further evaluate analytical sensitivity.

In terms of throughput, we were able to assess the performance of RespiV of up to 87 samples per run. With a single MP96 (1 h) and PCR plate set-up during extraction, combined with one thermal cycler (2 h), up to 957 samples can be tested and reported in 24 h. With two thermal cyclers and a coordinated approach, up to 1,827 samples can be tested in 24 h. In contrast, our testing capacity with RP2.1+ fully utilizing 6 modules is 192 samples in 24 h. The key point of difference is the test turnaround time for one sample with RP2.1+ (45 min). However, the test number threshold at which a 6‑module BioFire system becomes slower in overall throughput than RespiV, when measured from sample receipt to delivery of all results, is approximately 32 samples, requiring around 4 h to complete. This is where RespiV has a strong advantage in coping with an influx of samples or surge testing should it be necessary, reporting most of the common respiratory pathogens with a high degree of laboratory efficiency. To further enhance throughput, the manufacturer has recently presented a 384-well version of the RespiV assay, combining the KingFisher Flex System (ThermoFisher) with automated data analysis, with a throughput of 372 samples in a single run (17).

In terms of targets, although RespiV has the additional claim of EV-specific detection, RP2.1+ includes four bacterial pathogens and the additional seasonal coronaviruses. Based on the results of this study, the seasonal coronavirus detection is a significant advantage compared to RespiV, with many additional detections as the sole pathogen, which would otherwise be reported as negative if RespiV were the front-line test. As such, a non-differentiated seasonal coronavirus detection using one or more of the available reporter dyes (CY5 and/or CY5.5) at 76°C (Fig. 2) would complete the viral testing strategy for these common causes of ARI. Despite the lack of some targets compared to RP2.1+, the RespiV assay has several advantages other than throughput. These include providing quantitative information about SARS-CoV-2 viral load by our calibration to the IS, or the analysis of *C_q_* values for other viruses. *C_q_* values for RP2.1+ can be accessed through the Cp Viewer component of the BioFire FIREWORKS Software (bioMérieux). However, according to the software’s terms of use, these *C*_*q*_ values are not permitted for clinical decision-making. This restriction applies even though RP2.1+ was successfully calibrated to the SARS-CoV-2 IS in this study. As such, the *C*_*q*_ values and regression curves for both RP2.1+ targets were not presented in the supplemental material. Additionally, the use of *C_q_* is an essential tool in the laboratory for the monitoring of diagnostic test performance. Apart from the qualitative detection of targets to quality control RP2.1+ assay kits, a finer resolution of lot-to-lot variation in test performance cannot be performed unless *C_q_* values or quantitation standards are used. For example, loss in analytical sensitivity leading to variations in the lower limit of detection for one or more targets in the RP2.1+ assay may not be detected using qualitative controls. This may result in false negative results impacting patient care. In contrast, *C_q_* values and quantitative analysis of the RespiV assay guarantee quality assurance from lot to lot and run to run.

Although we tested a considerable number of samples, our study has some limitations. While the retrospective design allowed efficient batch testing, a prospective head-to-head comparison would better control for sample degradation and timing bias—samples in our study were first tested prospectively with RP2.1+, then frozen and subsequently thawed for re-testing with RespiV. After this, samples were frozen again, and any discordant cases were thawed again for additional discordant analysis. Additionally, cost analysis, multi-target analysis, and multiplex interference studies (e.g., checkerboard experiments) would provide further insight into assay performance. Future evaluations should include lower limit of detection studies for other targets, particularly FluA, and independent confirmation of EV specificity. However, we did note RespiV’s ability to detect down to 1.70 log_10_ IU/mL for SARS-CoV-2, comparable to Xpert and more sensitive than RP2.1+.

This study represents the first evaluation of RespiV, a multiplex assay that detects multiple targets within each optical channel using multiple probes that generate fluorescent signals at distinct temperatures. As we have shown, this enhanced multiplexing capability is well-suited for high-throughput syndromic testing of multiple pathogens. Similarly, Roche Diagnostics has recently developed cobas Respiratory Flex (RespFlex), which employs temperature activation of signal technology to simultaneously detect and differentiate up to 12 viral respiratory pathogens (18). While RespFlex includes similar targets to RespiV, it also covers the common coronaviruses—229E, HKU1, NL63, and OC43. Moreover, RespFlex is a sample-to-answer assay compatible with the high-throughput cobas 6800/8000 systems, as well as the compact cobas 5800 system. Considering the extended targets and workflow advantage, our laboratory is conducting a comparative study during the 2025 influenza season, evaluating RP2.1+, RespFlex, and the updated version of RespiV, which now includes the common coronaviruses. These emerging multiplexing technologies are advancing the capabilities of traditional thermal cyclers and platforms that utilize them, narrowing the gap in target coverage compared to other rapid multi-marker assays like RP2.1+, although RP2.1+ maintains the advantage of rapid result turnaround. The inclusion of common bacterial targets in these assays would further enhance their diagnostic utility. A synergistic testing strategy—offering both rapid and high-throughput assays with overlapping viral and bacterial targets—provides optimal flexibility and clinical value for diagnosing ARI.

## SUPPLEMENTAL MATERIAL

Supplemental material spectrum.00019-26-s0001.xlsx Data; Tables S1 and S2; Fig. S1.

ASM does not own the copyrights to Supplemental Material that may be linked to, or accessed through, an article. The authors have granted ASM a non-exclusive, world-wide license to publish the Supplemental Material files. Please contact the corresponding author directly for reuse.
